# A ring-shaped gas sign: a case of the accidental ingestion of a press-through package

**DOI:** 10.1007/s12328-024-02060-4

**Published:** 2024-11-12

**Authors:** Dai Kubota, Yosuke Tsuji, Yoku Hayakawa, Mitsuhiro Fujishiro

**Affiliations:** 1 Next-Generation Endoscopic Computer Vision, Graduate School of Medicine, The University of Tokyo, 7-3-1, Hongo, Bunkyo-Ku, Tokyo, 113-8655 Japan; 2 Department of Gastroenterology, Graduate School of Medicine, The University of Tokyo, 7-3-1, Hongo, Bunkyo-Ku, Tokyo, 113-8655 Japan

**Keywords:** Foreign body, Chest X-ray, Computed tomography

## Abstract

This case report presents a common but instructive clinical scenario of accidental ingestion of a press-through package. Despite an initial negative chest X-ray and mild symptoms, the diagnosis was confirmed with additional computed tomography. The patient was eventually went through the successful endoscopic removal of the press-through package. Furthermore, a retrospective re-reviewing of the X-ray revealed a faint ring-shaped gas sign, characteristic of press-through package ingestion. This case underscores the intractableness to diagnosis of accidental ingestion of press-through package by only X-rays in real time and the potential role of computed tomography in ensuring timely diagnosis and treatment.

## Introduction

Foreign body ingestion in adults is an emergency condition encountered in the emergency department. It has often occurred in patients with underlying factors such as mental disorders, developmental delays, dementia, or alcoholism [1–3]. Press-through packages (PTPs) are the common sharp objects which are ingested accidentally. One Japanese study reported that PTPs accounted for 33.5% of foreign body ingestion cases [4]. Data on accidental PTP ingestion in Japan are limited and the overall incidence remains poorly defined. According to the Japan Council for Quality Health Care, 71 cases were recorded between 2013 and 2020, with over half of these incidents involving individuals aged 70 and older. Importantly, incidents occurring in home settings are not captured in this data, indicating that the actual number of cases may be considerably higher. In this case, the initial suspicion of PTP ingestion was not confirmed by chest X-ray and the patient’s symptoms were so mild that she was almost sent home. However, the diagnosis was eventually made easily by adding computed tomography (CT), and the patient was treated by endoscopic foreign body removal. Furthermore, upon re-reviewing the chest X-ray taken before, a slight ring-shaped gaseous area was observed between the pill and the PTP. We report a typical but instructive case of accidental ingestion of PTP.

## Case report

**Fig. 1:**
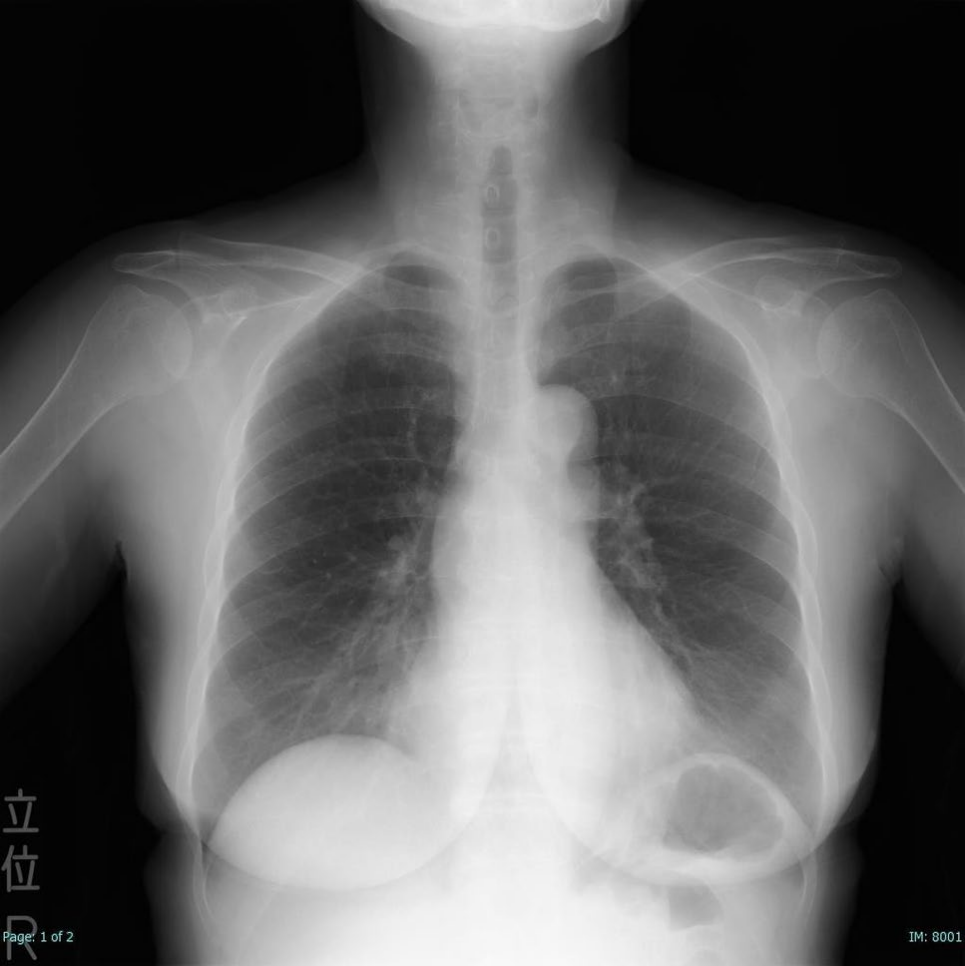
No foreign bodies were observed upon initial examination of the chest X-ray

**Fig. 2:**
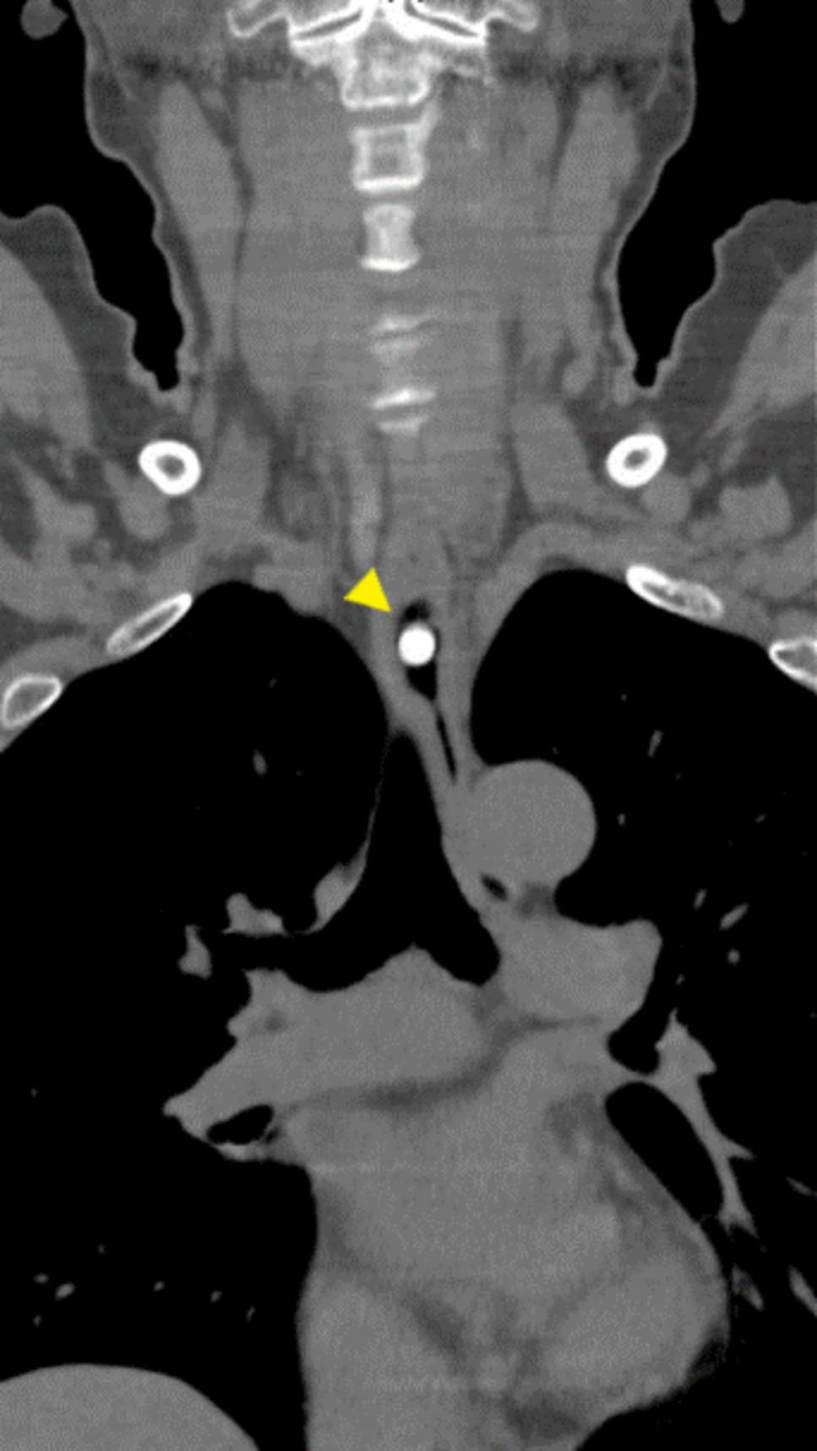
Computed tomography revealed a round, high-density area in the middle thoracic esophagus (yellow arrow)

**Fig. 3:**
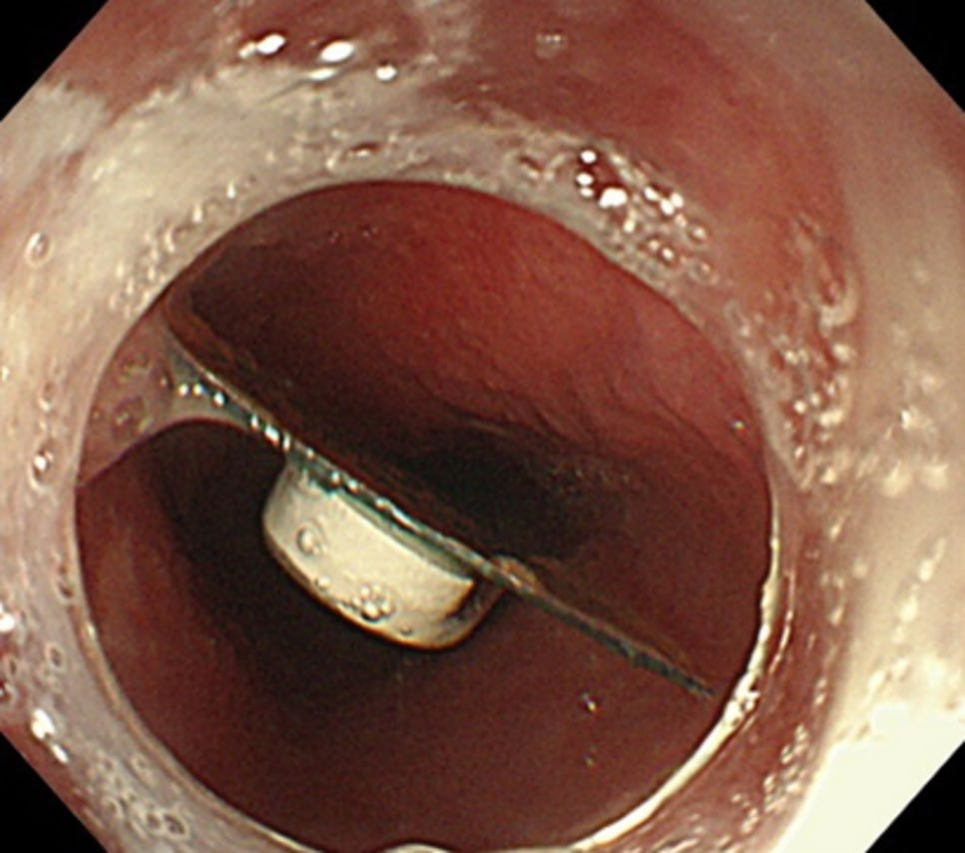
Lodging of the press-through package in the middle esophagus

**Fig. 4:**
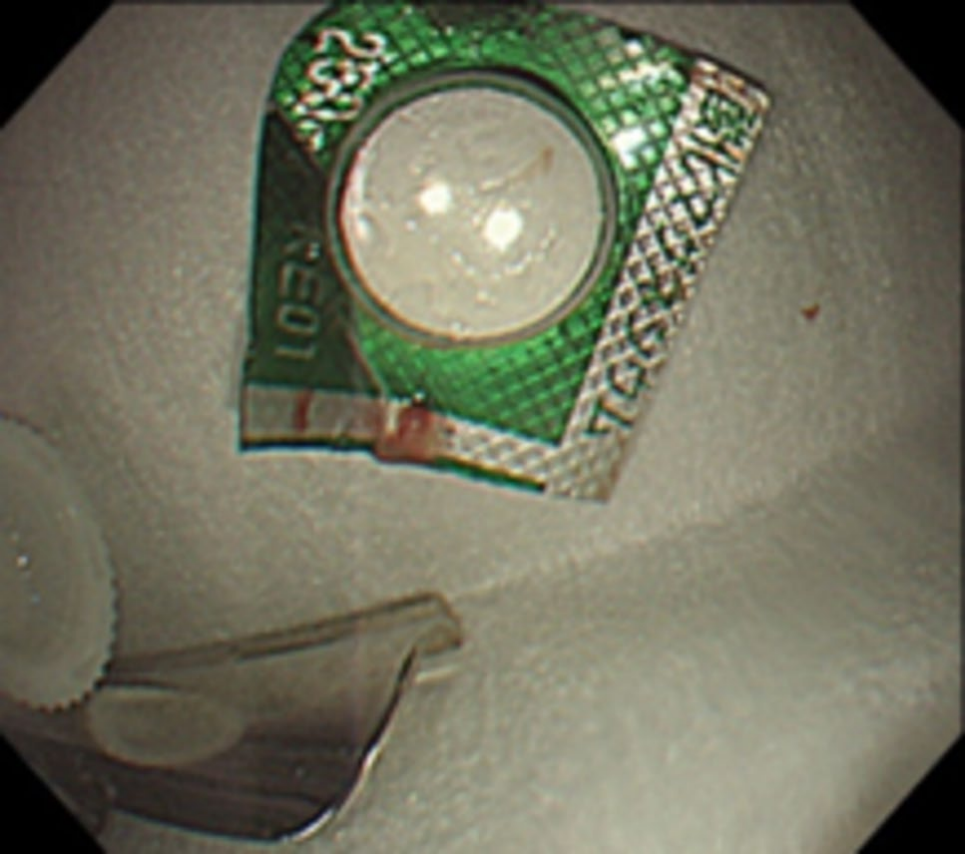
Removal of the press-through package using forceps

**Fig. 5:**
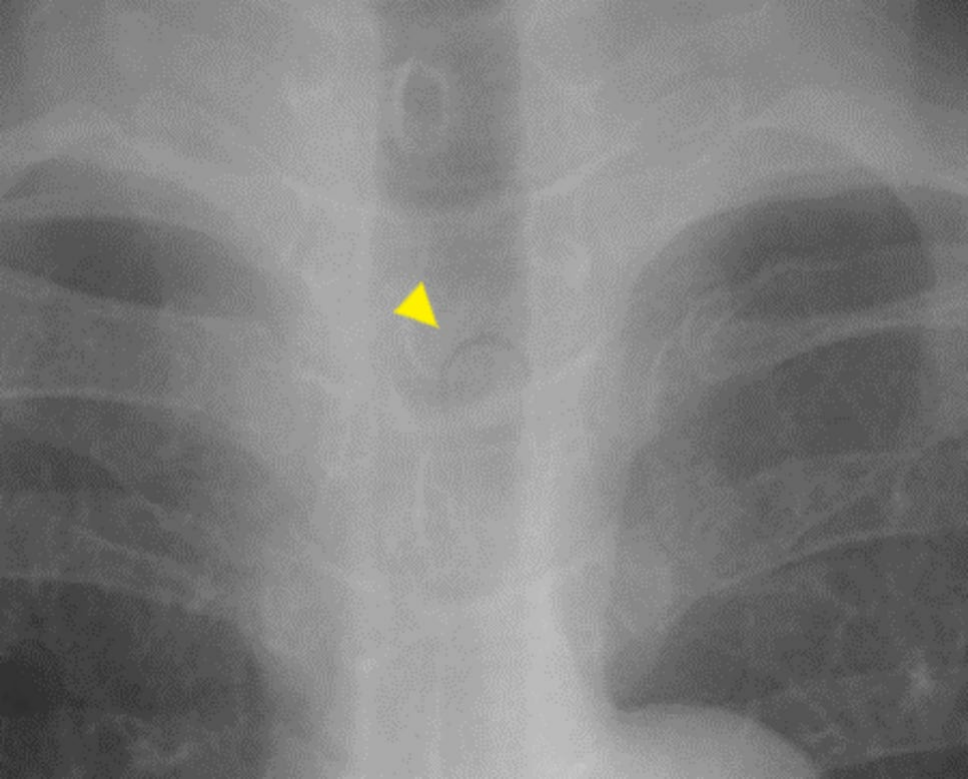
Upon reviewing the initial X-ray image, a slight ring-shaped gas sign between the pill and press-through package was observed (yellow arrow) (Enlarged image of Fig. 1)

A 66-year-old woman with multiple system atrophy presented to our emergency department with a mild sore throat after taking her medication following lunch, suspecting accidental ingestion of a PTP. She had gait disturbance, dysarthria, dysphagia, and required nursing care for activities of daily living. Her medications, managed by a caregiver who cut individual tablets from the PTP with scissors, included taltirelin hydrate, mirabegron, and magnesium oxide. However, the patient’s recollection of the ingestion was unclear. The family searched the garbage bucket, attempting to determine ingestion by the number of empty PTPs and remaining medication. However, the exact count before symptom onset was unknown due to self-adjusted dosing of mirabegron and magnesium oxide, rendering the assessment inconclusive. Vital signs were normal and no significant findings were observed during the physical examination, with only mild throat discomfort reported at the emergency department. The electrocardiogram showed no abnormalities. Given the medical history and clinical findings, PTP ingestion was suspected. Chest X-ray was taken and reviewed by several gastroenterologists; no obvious foreign body was identified (Fig. 1). One hour after admission, the patient felt that the throat discomfort persisted, although it seemed to have improved. Globus syndrome was provisionally considered, and both the patient and family were convinced that there was no PTP ingestion, contemplating home observation. However, due to the persistence of minor symptoms, the possibility of a missed diagnosis on the X-ray was considered and CT was added. As a result, CT revealed a clear, round, high-density area in the middle thoracic esophagus (Fig. 2). An emergency esophagogastroduodenoscopy (EGD) was performed for further investigation of the foreign body seen by CT, and a PTP containing a pill was detected in the middle esophagus (Fig. 3), which was subsequently removed using forceps (Fig. 4). The ingested PTP contained magnesium oxide and was made of polyvinyl chloride (PVC). The patient had an intraoperative minor esophageal tear that required hospitalization for follow-up, while the mild throat discomfort completely disappeared immediately after the procedure. After 1 day, the absence of symptom recurrence was confirmed, along with normal blood tests and no evident mediastinal emphysema on X-ray. The patient and family were instructed to avoid cutting PTPs into individual tablets with scissors in the future and the patient was discharged. Later, a radiologist reviewed the X-ray image taken at the time of the emergency department visit and pointed out a slight ring-shaped gaseous area in the mid-chest, thought to be air within the PTP (Fig. 5).

## Discussion

This case represents a common scenario of accidental ingestion of PTP but serves as an instructive case for not only gastroenterologists but also all primary care doctors. The chest X-ray did not reveal any abnormalities and the patient’s history and symptoms were unclear, the patient had almost been sent home. However, the diagnosis was made with an additional CT, and PTP removal was achieved. Delayed diagnosis could have resulted in gastrointestinal perforation due to the PTP. While diagnosing PTP with CT is typical, retrospectively, the chest X-ray also faintly showed a ring-shaped gas sign characteristic of PTP ingestion. This case suggests that a careful review of the X-ray could have led to a quicker diagnosis. Although other cases published describing the visualization of PTP on neck or abdominal X-ray [5, 6], to our knowledge, this is the first report capturing a ring-shaped gas sign on a frontal view of the chest X-ray in the real patient. Nevertheless, as demonstrated in this case, detecting a PTP in the esophagus using only X-rays can be challenging in a busy emergency department setting, and adding a CT should have been considered first. The utilization of overtubes has been shown to enhance the safety of PTP removal procedures[7]. In this case, the CT scan indicated that the PTP was relatively large, necessitating the initial insertion of an overtube. Despite multiple attempts to retract the PTP into the overtube using forceps and a snare, the suboptimal angle resulted in repeated minor esophageal lacerations at the site of the second natural constriction. Recent literature suggests that employing a large-caliber, soft oblique cap can reduce mucosal injury during foreign body retrieval [4]. We propose that if this technique had been utilized, it is conceivable that the PTP could have been effectively retracted into the cap, potentially preventing esophageal lacerations at the constriction site. The types of foreign body ingestion vary by region, with fish bones being the most common type reported in adults in some studies [8]. In Japan, PTPs account for 30% of foreign body ingestion causing the highest proportion [4]. Currently, efforts in Japan to prevent PTP ingestion include eliminating perforations in PTP sheets to discourage cutting into individual tablets and active awareness campaigns through posters by the National Consumer Affairs Center of Japan in collaboration with the Ministry of Health, Labour and Welfare. However, some patients and caregivers unaware of the risks still manage medication by cutting individual tablets [9]. PTP ingestion can sometimes lead to serious complications such as gastrointestinal perforation, making early diagnosis and removal crucial. PTP ingestion often occurs in patients with poor general health, and CT is considered useful when suspecting PTP ingestion [10]. Although some case reports suggest that X-rays can be useful for diagnosing PTP ingestion [5, 6], others report a sensitivity of 0% [10]. Polypropylene (PP) and PVC are commonly utilized materials in PTP packaging. While only PP is radiolucent on CT, both PP and PVC are radiolucent on X-ray. Consequently, if the orientation remains unchanged, the ring-shaped gas sign, indicative of air trapped between the tablet and the PTP sheet, can be detected on X-ray irrespective of the material. Looking back at this case, the diagnosis could have been made with X-ray alone, but detecting a circular gas shadow like this case would have been difficult unless the PTP was facing forward. The diagnostic utility of the ring-shaped sign in cases of accidental PTP ingestion remains indeterminate. However, it may serve as a valuable indicator in primary care settings where only X-ray imaging is available. However, it would be extremely difficult to diagnose PTP ingestion by X-ray alone in a generally busy emergency room. Therefore, in cases where PTP ingestion cannot be ruled out, emergency examinations such as CT or EGD should be considered first. If these procedures are difficult in your institutions, although X-rays may be considered first, early transfer to a higher-level medical institution should also be considered.
